# Anti-inflammatory compounds from the mangrove endophytic fungus *Amorosia* sp. SCSIO 41026

**DOI:** 10.3389/fmicb.2022.976399

**Published:** 2022-08-29

**Authors:** Xue Ren, Chunmei Chen, Yuxiu Ye, Ziying Xu, Qingliang Zhao, Xiaowei Luo, Yonghong Liu, Peng Guo

**Affiliations:** ^1^Capital Institute of Pediatrics, Beijing, China; ^2^CAS Key Laboratory of Tropical Marine Bio-resources and Ecology/Guangdong Key Laboratory of Marine Materia Medica, South China Sea Institute of Oceanology, Chinese Academy of Sciences, Guangzhou, China; ^3^University of Chinese Academy of Sciences, Beijing, China; ^4^Institute of Marine Drugs, Guangxi University of Chinese Medicine, Nanning, China; ^5^State Key Laboratory of Molecular Vaccinology and Molecular Diagnostics, Center for Molecular Imaging and Translational Medicine, Department of Laboratory Medicine, School of Public Health, Xiamen University, Xiamen, China; ^6^Southern Marine Science and Engineering Guangdong Laboratory, Guangzhou, China

**Keywords:** marine fungi, *Amorosia* sp., chlorinated compounds, anti-inflammatory activity, acute lung injury mice

## Abstract

Three new chlorinated compounds, including two propenylphenol derivatives, chlorophenol A and B (**1** and **2**), and one benzofuran derivative, chlorophenol C (**3**), together with 16 known compounds, were isolated from the mangrove endophytic fungus *Amorosia* sp. SCSIO 41026. 7-Chloro-3,4-dihydro-6,8-dihydroxy-3-methylisocoumarine (**4**) and 2,4-dichloro-3-hydroxy-5-methoxy-toluene (**5**) were obtained as new natural products. Their structures were elucidated by physicochemical properties and extensive spectroscopic analysis. Compounds **1**, **4**, **7**, **9**, **13**, **15**, **16**, and **19** possessed inhibitory effects against the excessive production of nitric oxide (NO) and pro-inflammatory cytokines in lipopolysaccharide (LPS)-challenged RAW264.7 macrophages without obvious cytotoxicity. Moreover, 5-chloro-6-hydroxymellein (**13**) further alleviated the pathological lung injury of LPS-administrated mice and protected RAW264.7 macrophages against LPS-induced inflammation through PI3K/AKT pathway *in vivo*. Our research laid the foundation for the application of compound **13** as a potential anti-inflammatory candidate.

## Introduction

As an adaptive response, inflammation is triggered by harmful stimuli and conditions that prompt the host to produce pro-inflammatory cytokines including interferon, interleukin, chemokines, and tumor necrosis factor against pathogens (Al-Lamki et al., 2001). A controlled inflammatory response is generally thought to be beneficial, in providing protection against infection for example, but it can become harmful or even fatal if it is maladjusted (e.g., rheumatoid arthritis, systemic infection, and septic shock). Nitric oxide (NO) is synthesized by inducible nitric oxide synthase type 2 (NOS-2) in many cells involved in immunity and inflammation. Studies have shown that overproduction of NO is rapidly oxidized to reactive nitrogen oxides (RNOs; Coleman, 2001), which are able to modify key signal molecules such as kinases and transcription factors. In addition, RNOs restrain crucial enzymes in mitochondrial respiration, resulting in intra-cellular ATP depletion. In addition, a high concentration of NO inhibited the activity of antigen-presenting cells and T-cell proliferation (Kim et al., 2002; Hortelano et al., 2003). Therefore, targeting the reduction of these pro-inflammatory mediators can be an effective way to control and prevent chronic inflammatory diseases to some extent.

Lipopolysaccharide (LPS) is a virulence factor of the outer membrane of gram-negative bacterial cell wall, of which lipid A is the immunologically active component and can cause an inflammatory response. LPS induces the translocation of the transcription factor NF-*κ*B into the nucleus by binding to cells expressing toll-like receptor 4 (TLR4), resulting in dephosphorylation of phosphorylated interferon regulatory factor 3 (IRF3) and stimulating the production of type I interferons, which causes subsequent inflammatory response (Yoo et al., 2014; Peerapornratana et al., 2019; Zhuo et al., 2019). Although NF-*κ*B signaling has been historically considered a classic LPS-induced inflammatory pathway, it is of great significance to enthusiastically search for the in-depth understanding of the inflammatory response mechanism and the development of target drugs.

Halogenated organic molecules are generally rare in nature and could simply be classified as fluorinated, chlorinated, brominated, or iodinated compounds (Wang et al., 2013). Over 5,000 halogenated natural products had been reported as of 2011 with structural and functional diversity (Gribble, 2015; Zeng and Zhan, 2019). As a key part of halogenated molecules, chlorinated compounds have demonstrated a broad range of remarkable activities (Gribble, 2015), including antibacterial activity (Bracegirdle et al., 2021; Saetang et al., 2021) and anticancer activity (Luo et al., 2018). Recently, three new natural chlorinated 3-phenylpropanoic acid derivatives were found with significant and selective inhibitory activities toward *Escherichia coli* and *Staphylococcus aureus* (Shaala et al., 2020).

**Figure 1:**
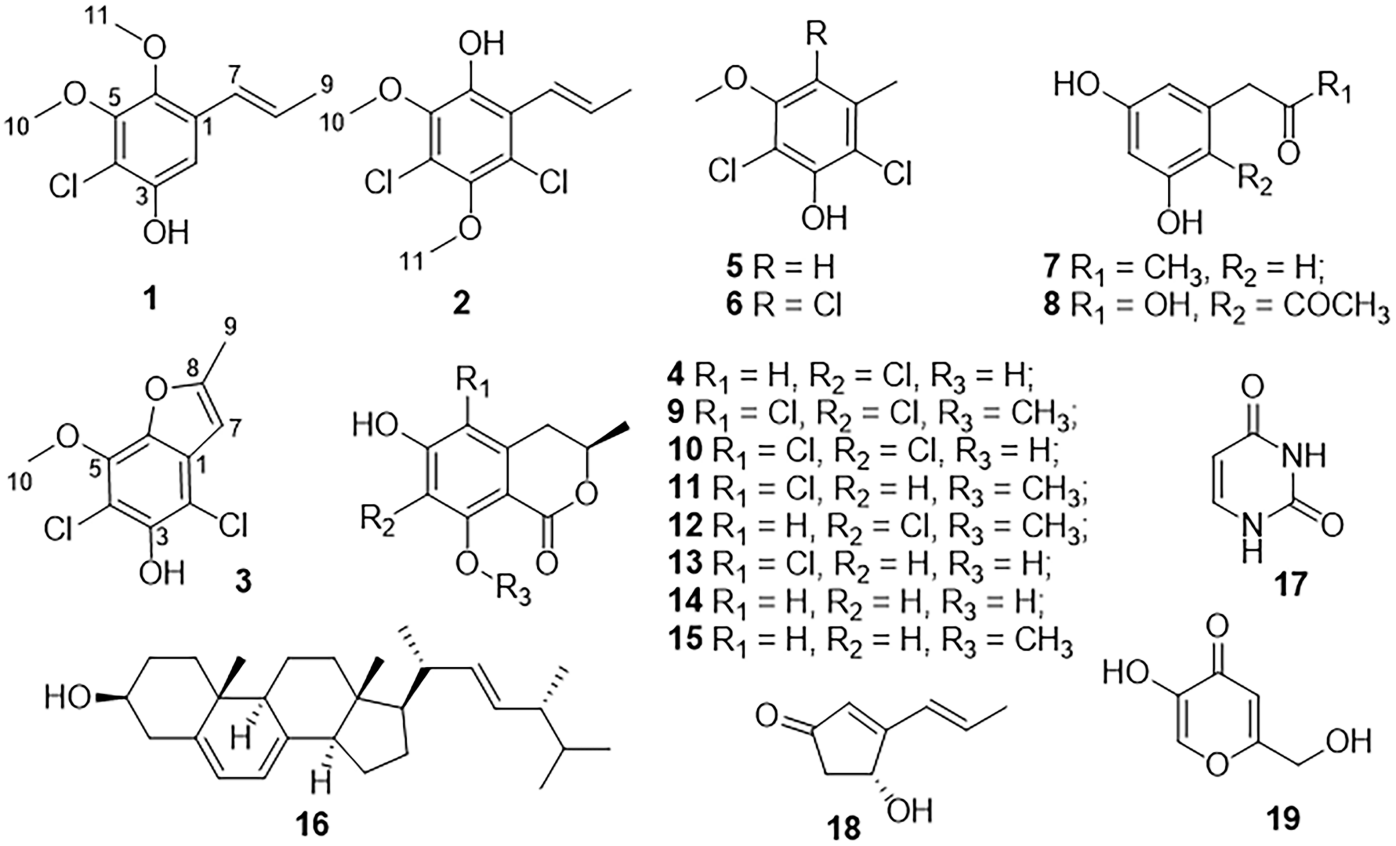
Structures of compounds **1**–**19**.

Naturally occurring chlorinated compounds were mainly isolated from the marine environment and microorganisms (Wang et al., 2013, 2021a,b). Mangrove forests are complex ecosystems with the frequent tide, which contained a high concentration of chloride ions. Therefore, mangrove-derived fungi are proved to be a promising source of novel and unique chlorine-containing bioactive secondary metabolites (Xu, 2015; Chen et al., 2021a). As part of our continuous efforts to discover structurally novel and bioactive secondary metabolites from mangrove endophytic fungi (Chen et al., 2021b,d), three new chlorine-containing compounds, chlorophenol A − C (**1**–**3**), and two new natural products (**4** and **5**), together with 14 known compounds, were isolated from the mangrove-derived fungus *Amorosia* sp. SCSIO 41026. Herein, the isolation, structure elucidation, and biological effects of compounds **1**–**19** (Figure 1) are described.

## Materials and methods

### General experimental procedures

The UV and IR spectra were recorded on a Shimadzu UV-2600 PC spectrometer (Shimadzu) and an IR Affinity-1 spectrometer (Shimadzu). Optical rotations were determined with an Anton Paar MPC 500 polarimeter. HRESIMS spectra were recorded with a Bruker maXis Q-TOF mass spectrometer. The NMR spectra were recorded on a Bruker Avance-500 or 700 spectrometers (Bruker BioSpin International AG, Switzerland) using tetramethylsilane as internal standard, and chemical shifts were recorded as *δ*-values. Semipreparative high-performance liquid chromatography (HPLC) was performed on the Hitachi Primaide with a DAD detector, using an ODS column (YMC-pack ODS-A, 10 mm × 250 mm, 5 μm). Thin-layer chromatography analysis (TLC) and column chromatography (CC) were performed on plates precoated with silica gel GF254 (10–40 μm) and over silica gel (200–300 mesh; Qingdao Marine Chemical Factory) and Sephadex LH-20 (Amersham Biosciences, Uppsala, Sweden), respectively. Spots were detected on TLC (Qingdao Marine Chemical Factory) under 254 nm UV light. All solvents employed were of analytical grade (Tianjin Fuyu Chemical and Industry Factory).

### Fungal material

The endophytic fungal strain SCSIO 41026 was isolated from the leaf of *Avicennia marina* (Forsk.) Vierh. collected from the mangrove wetland in Zhanjiang, Guangdong province, China (21.235° N, 110.451° E). The strain was stored on MB agar (malt extract 15 g, sea salt 10 g, agar 16 g, H_2_O 1 l, and pH 7.4–7.8) slants at 4°C and deposited at the Key Laboratory of Tropical Marine Bio-resources and Ecology, Chinese Academy of Sciences. The ITS1-5.8S-ITS4 sequence region (GenBank accession No. OL826791) of strain SCSIO 41026 was amplified by PCR. The DNA sequencing showed it shared significant homology to the sequence of *Amorosia littoralis* (accession No. AM292047.1), so it was designated as *Amorosia* sp. SCSIO 41026.

### Fermentation and extraction

A large-scale fermentation of fungal strain SCSIO 41026 was incubated at 28°C for 14 days on a rotating shaker (180 rpm) in 1 L conical flasks containing the liquid medium (300 ml/flask) composed of mannitol (2%), yeast extract (0.3%), MgSO_4_·7H_2_O (0.03%), sodium glutamate (1%), glucose (1%), KH_2_PO_4_ (0.05%), sucrose (2%), corn steep liquor (0.1%), artificial sea salt (3.3%), and tap water after adjusting its pH to 7.5. The whole fermented broth (33 L) was overlaid with EtOAc, sonicated for 15 min, and extracted with EtOAc three times to afford a brown extract (22.0 g).

### Isolation and purification

The organic extract was subjected to silica gel CC using step gradient elution with petroleum ether/CH_2_Cl_2_ (0–100%, *v/v*) and CH_2_Cl_2_/CH_3_OH (0–100%, *v/v*) to obtain 10 subfractions (Frs. 1–10) based on TLC properties.

Fraction 1 was separated by semipreparative reverse-phase HPLC (74% CH_3_OH/H_2_O, 2 ml/min) to afford **5** (4.5 mg, *t*_R_ = 12.5 min), **3** (5.9 mg, *t*_R_ = 17.7 min), **6** (7.8 mg, *t*_R_ = 26.0 min), and **2** (8.2 mg, *t*_R_ = 28.8 min). Fraction 2 was also subjected to semipreparative HPLC eluting with 62% CH_3_OH/H_2_O to give **10** (13.3 mg, *t*_R_ = 22.3 min) and **1** (2.6 mg, *t*_R_ = 27.5 min). Fraction 3 was divided into a white solid (16, 51.5 mg) and a methanol solution (Fr.3–2) by filtration. Fr. 3–2 was further subjected to a Sephadex LH-20 column eluting with CH_3_OH, followed by semipreparative HPLC (58% CH_3_OH/H_2_O, 2 ml/min) to afford **12** (83.5 mg, *t*_R_ = 11.5 min), **14** (20.1 mg, *t*_R_ = 13.2 min), **4** (10.8 mg, *t*_R_ = 16.8 min), **9** (17.6 mg, *t*_R_ = 18.8 min), and **13** (10.6 mg, *t*_R_ = 22.5 min). Fraction 6 was purified by semipreparative HPLC (35% CH_3_CN/H_2_O, 2 ml/min) to offer **18** (6.7 mg, *t*_R_ = 11.3 min), **15** (7.1 mg, *t*_R_ = 15.0 min), and **11** (6.3 mg, *t*_R_ = 22.0 min). **7** (6.2 mg, *t*_R_ = 16.0 min) was obtained from fraction 8 by semipreparative HPLC eluting with 15% CH_3_CN/H_2_O (2 ml/min). Fraction 10 was separated by semipreparative HPLC (30% CH_3_OH/H_2_O, 2 ml/min) to afford **17** (6.3 mg, *t*_R_ = 7.0 min), **19** (70.5 mg, *t*_R_ = 8.5 min), and **8** (11.0 mg, *t*_R_ = 17.5 min).

### Spectroscopic data of compounds

The specifications are as follows:

Chlorophenol A (**1**): colorless oil; UV (CH_3_OH) *λ*_max_ (log *ε*) 206 (4.04), 254 (3.53), and 320 (3.29) nm; IR (film)*ν*_max_ 3,385, 2,929, 1,680, 1,458, 1,411, 1,205, 1,141, 1,070, 1,012, 844, 802, and 725 cm^−1^; ^1^H and ^13^C NMR data, Table 1; and HRESIMS *m*/*z* 227.0476 [M − H]^−^ (calcd for C_11_H_12_ClO_3_^−^, 227.0480).

Chlorophenol B (**2**): colorless oil; UV (CH_3_OH) *λ*_max_ (log *ε*) 211 (4.12), 261 (3.62), and 298 (3.33) nm; IR (film)*ν*_max_ 3,394, 2,937, 1,680, 1,454, 1,408, 1,348, 1,205, 1,143, 1,083, 985, 798, and 723 cm^−1^; ^1^H and ^13^C NMR data, Table 1; and HRESIMS *m*/*z* 261.0092 [M − H]^−^ (calcd for C_11_H_11_Cl_2_O_3_^−^, 261.0091).

Chlorophenol C (**3**): brown oil; UV (CH_3_OH) *λ*_max_ (log *ε*) 216 (4.08), 261 (3.76), and 292 (3.19) nm; IR (film)*ν*_max_ 2,926, 2,854, 1,683, 1,472, 1,437, 1,375, 1,339, 1,209, 1,142, 1,005, 980, 947, 910, and 723 cm^−1^; ^1^H and ^13^C NMR data, Table 1; and HRESIMS *m*/*z* 244.9786 [M − H]^−^ (calcd for C_10_H_7_Cl_2_O_3_^−^, 244.9778).

**Table 1:** ^1^H (500 MHz) and ^13^C (125 MHz) NMR data for **1**–**3** (*δ* in ppm, DMSO-*d*_6_).

Pos.	**1**		**2**		**3**	
	*δ*_C_ type	*δ*_H_ (*J* in Hz)	*δ*_C_ type	*δ*_H_ (*J* in Hz)	*δ*_C_ type	*δ*_H_ (*J* in Hz)
1	129.8, C		122.8, C		128.3, C	
2	106.9, CH	6.83 (s)	122.4, C		110.4, C	
3	150.0, C		144.5, C		145.5, C	
4	113.0, C		120.0, C		104.9, C	
5	150.0, C		143.3, C		139.6, C	
6	143.1, C		146.1, C		138.7, C	
7	124.3, CH	6.53 (dd, 16.0, 1.5)	123.1, CH	6.42 (dd, 16.0, 1.5)	101.7, CH	6.61 (d, 1.0)
8	127.3, CH	6.19 (dq, 16.0, 6.5)	133.5, CH	6.58 (dq, 16.0, 6.5)	157.7, C	
9	18.7, CH_3_	1.88 (dd, 6.5, 1.5)	19.6, CH_3_	1.89 (dd, 6.5, 1.5)	13.8, CH_3_	2.47 (d, 1.0)
10	60.5, CH_3_	3.80 (s)	60.4, CH_3_	3.73 (s)	60.6, CH_3_	4.12 (s)
11	61.1, CH_3_	3.68 (s)	60.7, CH_3_	3.72 (s)		
OH		9.97 (s)		9.63 (s)		9.75 (s)

### X-ray crystallographic analysis

The clear light colorless crystals of **4** and **12** were obtained in MeOH by slow evaporation. Crystallographic data for the structures have been deposited in the Cambridge Crystallographic Data Centre. Copies of the data can be obtained, free of charge, on application to CCDC, 12 Union Road, Cambridge CB21EZ, United Kingdom [fax: +44(0)-1,223–336,033 or e-mail: deposit@ccdc.cam.ac.uk].

Crystal data for **4**: 2C_10_H_9_ClO_4_·2CH_3_OH, *M*_r_ = 521.33, crystal size 0.18 × 0.09 × 0.08 mm^3^, monoclinic, *a* = 7.17000 (10) Å, *b* = 15.1731 (2) Å, *c* = 10.73390 (10) Å, *α* = 90°, *β* = 90.9170 (10)°, *γ* = 90°, *V* = 1167.60 (2) Å3, *Z* = 2, *T* = 100.00 (10) K, space group *P*2_1_, *μ*(Cu K*α*) = 1.54184 mm^−1^, *D*_calc_ = 1.483 g/cm^3^, 11,652 reflections measured (8.238° ≤ 2Θ ≤ 148.702°), and 4,588 unique (*R*_int_ = 0.0251, *R*_sigma_ = 0.0301). The final *R*_1_ values were 0.0274 [*I* > 2σ(*I*)]. The final *wR*(*F*^2^) values were 0.0745 [*I* > 2σ(*I*)]. The final *R*_1_ values were 0.0279 (all data). The final *wR*(*F*^2^) values were 0.0748 (all data). The goodness of fit on *F*^2^ was 1.069. The flack parameter was 0.005 (5) (CCDC 2133225).

Crystal data for **12**: 4C_11_H_11_ClO_4_, *M*_r_ = 970.59, crystal size 0.19 × 0.15 × 0.04 mm^3^, triclinic, *a* = 7.5028 (2) Å, *b* = 10.4928 (4) Å, *c* = 14.5209 (4) Å, *α* = 105.964 (3)°, *β* = 90.259 (2)°, *γ* = 96.779 (2)°, *V* = 1090.57 (6) Å3, *Z* = 1, *T* = 100.00 (10) K, space group *P*1, *μ*(Cu K*α*) = 1.54184 mm^−1^, *D*c_alc_ = 1.478 g/cm^3^, 10,467 reflections measured (8.834° ≤ 2Θ ≤ 149.016°), and 5,572 unique (*R*_int_ = 0.0559, *R*_sigma_ = 0.0601). The final *R*_1_ values were 0.0551 [*I* > 2σ(*I*)]. The final *wR*(*F*^2^) values were 0.1339 [*I* > 2σ(*I*)]. The final *R*_1_ values were 0.0624 (all data). The final *wR*(*F*^2^) values were 0.1373 (all data). The goodness of fit on *F*^2^ was 1.062. The flack parameter was 0.000 (16) (CCDC 2133224).

### RAW264.7 **c**ell **c**ulture and LPS-induced **i**nflammation **c**ell **m**odel

RAW264.7 macrophages were obtained from Peking Union Medical College. Cells were maintained in Dulbecco’s modified Eagle’s medium (DMEM) with high glucose (Corning, Corning, NY, United States) supplemented with 10% (*v/v*) fetal bovine serum (FBS, Gibco, Grand Island, NY, United States), penicillin (100 U/ml), and streptomycin (100 mg/ml; Thermo Scientific, Waltham, MA, United States) in a 100% humidified incubator with 5% CO_2_ at 37°C. The culture medium was changed every 2 days.

For LPS induction, RAW264.7 cells were cultured in a 24-well plate at a density of 1 × 10^5^ cells/well (1 ml medium/well) overnight. After 24 h of exposure to 0.1 μg/ml LPS (#L2630, Merck, Shanghai, China) and being separately co-treated with the compounds (final concentration was maintained at 10 μM), the culture supernatant was collected to measure the production of NO, IL-6, TNF-*α*, and MCP-1, and cells were digested and harvested for RNA extraction.

### Cell viability assay

3 × 10^4^ cells/well (100 μl medium/well) were seeded in a 96-well plate overnight; then, they were exposed to DMEM supplemented with the compounds (final concentration was maintained at 10 μM) for 24 h, respectively. At the same time, the wells without cells were set as blank group and the drug-free cells as control group. Whereafter, 10 μl CCK-8 solution (#C0037, Beyotime Institute of Biotechnology, Shanghai, China) was added to each well and incubated at 37°C for 1 h. The absorbance was read at 450 nm with a microplate reader (Thermo, Waltham, MA, United States). Cell viability was calculated by the formula (experimental group absorbance value/control group absorbance value) × 100%.

### Nitric oxide production assay

Nitric oxide production was assessed by a Nitric Oxide Assay Kit (#S0021, Beyotime Institute of Biotechnology, Shanghai, China) according to the manufacturer’s instructions; in the meanwhile, L-NMMA was used as a positive control of compound treatment since it is the total NOS inhibitor. Briefly, after 24 h of LPS induction, 50 μl cell culture supernatant was transferred to another 96-well plate, and then, 50 μl Griess reagents I and Griess reagents II were successively added. The absorbance was read at 540 nm with a microplate reader. The calibration curve was contained in the Supplementary Figure 24.

### Cytokine detection

After 24 h of LPS induction, the cell culture supernatant was collected to measure the release of tumor necrosis factor *α* (TNF-*α*), interleukin 6 (IL-6), and monocyte chemoattractant protein 1 (MCP-1). Cytokine levels were quantified in the samples using an automatic ELISA (ELLA, protein simple).

### Quantitative real-time PCR analysis

Total RNA was isolated from cells using TRIzol® reagent (Ambion, Austin, Texas, United States) and was reverse-transcribed into cDNA using EasyScript® One-Step gDNA Removal and cDNA Synthesis SuperMix (TransGen Biotech, Beijing, China). Gene transcript levels of IL-6, TNF-*α*, MCP-1, IL-10, IL-4, and Arg-1 were quantified using the TransStart Top Green qPCR SuperMix (TransGen Biotech, Beijing, China) according to the manufacturer’s protocol. *β*-actin was used as housekeeping control. Primers used for quantitative PCR are listed in Supplementary Table 1.

### Animal experimental protocol

All animal experiments have been approved by the Medical Ethics Committee of Peking Union Medical College and comply with the regulations of the National Institutes of Health regarding the care and use of animals in research. The experiment programs on animals are shown in Supplementary Figure 28. Briefly, 6-week-old male BALB/c mice were purchased from Vital River Laboratories Co., Ltd. (Beijing, China). Animals were randomly divided into three groups (*n* = 6): the control group (Group B), the model group (5 mg/kg LPS; Group M), and LPS (5 mg/kg) + compound **13** (20 mg/kg) group (Group A). After 6 h of LPS administration, animals were killed for the bronchoalveolar lavage fluid (BALF) and lung tissue collection. Cytokine levels in BALF were detected by ELISA, and the upper left lungs from each group (*n* = 6 per group) were fixated with 4% buffered formalin solution and stained with hematoxylin and eosin (*H&E*).

### RNA sequencing

After 24 h of co-treatment of LPS and compound **13**, RAW264.7 macrophages were collected for RNA extraction and transcriptome sequencing. RNA sequencing was performed on the Annoroad Illumina-HiSeq platform. Differentially expressed genes were defined as values of *p* < 0.01. Pathway enrichment analysis using Gene Set Enrichment Analysis (GSEA).

### Western blot

The concentration was detected with BCA kit before total protein was extracted from RAW264.7 cells. The samples mixed with the loading buffer were separated on an 8% SDS-PAGE gel. After transferring to polyvinylidene fluoride membranes by electro-transfer, the membranes were blocked with 5% BSA at room temperature for 1 h. Next, the PVDF membranes were incubated with primary antibody overnight at 4°C and then incubated with peroxidase-conjugated secondary antibodies at room temperature for 1 h. The protein brands were captured in a gel imaging system. ImageJ was used to analyze the value of the brands.

### Statistical analysis

Statistical analyses were performed with GraphPad Prism (version 8.4.3). Independent Student’s *t*-tests were used to compare the means of numerical variables. Data were presented as the mean ± SD. Statistical significance was defined as *p* < 0.05.

## Results and discussion

### Structural characterization of these isolated compounds

**Figure 2:**
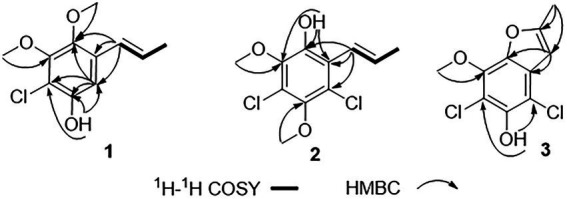
Key ^1^H-^1^H COSY and HMBC correlations of **1**–**3**.

Chlorophenol A (**1**) was obtained as a colorless oil and had the molecular formula C_11_H_13_ClO_3_ as determined by HRESIMS spectrum, which showed a cluster of deprotonated ion peaks at *m/z* 227.0476/229.0422 ([M − H] ^−^) with a ratio of 3:1, indicative of a monochlorinated compound (Luo et al., 2018). The 1D NMR (Table 1) and HSQC spectrum of **1** showed signals of five non-protonated sp^2^ carbons (*δ*_C_ 150.0, 150.0, 143.1, 129.8, and 113.0), one aromatic methine (*δ*_H/C_ 6.83/106.9), two olefinic methines (*δ*_H/C_ 6.53/124.3 and 6.19/127.3), two oxygenated methyls (*δ*_H/C_ 3.80/60.5 and 3.68/61.1), one methyl (*δ*_H/C_ 1.88/18.7), and one phenolic hydroxyl group (*δ*_H_ 9.97). The aforementioned data combined with five degrees of unsaturation (DOU) suggested that compound **2** presented a benzene ring skeleton, and the ^1^H-^1^H COSY correlations (Figure 2) between H-7/H-8 and H-8/H_3_-9 showed the existence of a propenyl group. Besides, the HMBC correlations (Figure 2) from H-7 to C-1, C-2, and C-6, and from H_3_-11 to C-6, revealed that the propenyl group and one methoxy group were located at C-1 and C-6, respectively. The HMBC correlations from 3-OH to C-2, C-3, and C-4 indicated the location of the phenolic hydroxyl group (C-3). The chemical shift of C-4 (*δ*_C_ 113.0) revealed that the chlorine substituent instead of the oxygenated methyl was attached at C-4. The other methoxy group was deduced to link with C-5 by the HMBC signal of H_3_-10/C-5 and the chemical shift of C-5 (*δ*_C_ 150.0). The aforementioned NMR characteristics showed great similarity to those of the co-isolated 2,4-dichloro-3-hydroxy-5-methoxy-toluene (**5**), which was reported as a synthetic chlorinated phenolic compound (Calam and Oxford, 1939). The main differences were the appearances of a propenyl group at C-1, a methoxy group at C-6, an aromatic hydrogen at C-2 in **1** instead of a methyl at C-1, an aromatic hydrogen at C-6, and a chlorine atom at C-2 in **5**, respectively. This deduction was further supported by the above 2D NMR data. Therefore, the structure of **1** was determined, as shown in Figure 1.

Chlorophenol B (**2**) was obtained as a colorless oil, and its molecular formula was deduced to be C_11_H_12_Cl_2_O_3_ by the HRESIMS peak at *m/z* 261.0092 [M − H] ^−^ (calcd. For C_11_H_11_Cl_2_O_3_^−^ 261.0091), suggesting five DOU. The 9:6:1 (*m/z* 261.0092/263.0065/265.0033) isotopic distribution pattern suggested the presence of two chlorine atoms (El-Kashef et al., 2020). The ^1^H, ^13^C (Table 1) NMR, and HSQC spectra of **2** showed signals of six non-protonated sp^2^ carbons (*δ*_C_ 146.1, 144.5, 143.3, 122.8, 122.4, and 120.0), two olefinic methines (*δ*_H/C_ 6.42/123.1 and 6.58/133.5), two methoxy groups (*δ*_H/C_ 3.73/60.4 and 3.72/60.7), one methyl (*δ*_H/C_ 1.89/19.6), and one phenolic hydroxyl group (*δ*_H_ 9.63). The above NMR data were highly similar to those of compound **1**. The main differences were the appearances of a chlorine atom at C-2, a methoxy group at C-3, and a hydroxyl group at C-6 in **2** instead of an aromatic hydrogen at C-2, a hydroxyl group at C-3, and a methoxy group at C-6 in **1**, respectively. This deduction was further supported by the 2D NMR data. The HMBC spectrum of **2** showed correlations from H-7 to C-1, C-2, and C-6, and from 6-OH to C-1 and C-6, which implied that the propenyl and the hydroxyl groups were located at C-1 and C-6, respectively. Besides, the HMBC correlations from 6-OH and H_3_-10 (*δ*_H_ 3.73) to C-5 (*δ*_C_ 143.3) suggested that the methoxy group (*δ*_H/C_ 3.73/60.4) was located at C-5. The chemical shift of C-2 (*δ*_C_ 122.4) indicated one of the chlorine atoms was attached to C-2. Besides, the HMBC correlation from H_3_-11 (*δ*_H_ 3.72) to C-3 (*δ*_C_ 144.5) verified that the remained one methoxy group (*δ*_H/C_ 3.72/60.4) was located at C-3. The above-detailed analysis of the NMR data allowed the assignment of all carbon and proton resonances of **2**. Consequently, the structure of **2** was elucidated, as shown in Figure 1.

Chlorophenol C (**3**) was obtained as brown oil. Analysis of the deprotonated molecule at *m/z* 244.9786 in the HRESIMS spectrum indicated a molecular formula of C_10_H_8_Cl_2_O_3_ (six DOU), and the 9:6:1 (*m/z* 244.9786/246.9759/248.9616) isotopic distribution also confirmed the presence of two chlorine atoms (El-Kashef et al., 2020). The ^1^H and ^13^C NMR data (Table 1) displayed signals of seven non-protonated sp^2^ carbons (*δ*_C_ 157.7, 145.5, 139.6, 138.7, 128.3, 110.4, and 104.9), one olefinic methine (*δ*_H/C_ 6.61/101.7), one oxygenated methyl (*δ*_H/C_ 4.12/60.6), one methyl (*δ*_H/C_ 2.47/13.8), and one phenolic hydroxyl group (*δ*_H_ 9.75). The above data and the HMBC correlations from 3-OH to C-2 and C-4, from H_3_-10 to C-5 implied that **3** was structurally related to the co-isolated 2,4-dichloro-3-hydroxy-5-methoxy-toluene (**5**), except the different substituents at C-1 and C-6. The above functional groups accounted for five DOU, and the remaining one DOU suggested that **3** possessed one ring. Furthermore, a methyl-furan ring linked to the benzene *via* C-1 and C-6 was deduced from the HMBC correlations from H-7 to C-1 and C-6, and from H_3_-9 to C-7 and C-8. Consequently, the structure of **3** was assigned as 4,6-dichloro-5-hydroxy-3-methoxy-10-methyl-benzofuran (Figure 1), which was similar to a synthetic compound, 4-chloro-7-hydroxy-5-methoxy-2-methyl-benzofuran (Lousberg and Tirilly, 1976). Compound **3** was finally characterized as shown in Figure 1 and was given the trivial name chlorophenol C.

**Figure 3:**
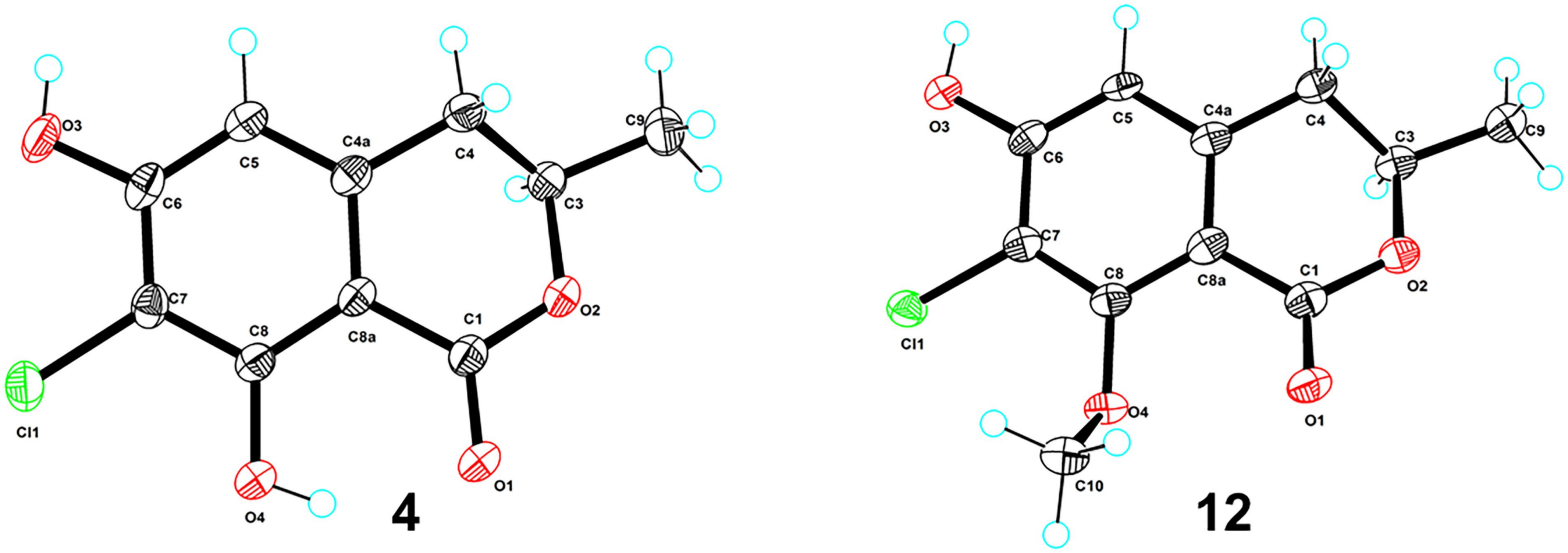
ORTEP diagrams of compounds **4** and **12**.

Both 7-chloro-3,4-dihydro-6,8-dihydroxy-3-methylisocoumarin (**4**; Henderson and Hill, 1982a,b) and 2,4-dichloro-3-hydroxy-5-methoxy-toluene (**5**; Calam and Oxford, 1939) were obtained as new natural products. Compound **4** was reported as a synthetic product (Henderson and Hill, 1982a) and has been suggested as an intermediate in the biosynthesis of cryptosporiopsinol-type secondary metabolites (Henderson and Hill, 1982a). Compound **5** was also known as a synthetic product without reported bioactive data (Calam and Oxford, 1939). Meanwhile, the other 14 known compounds were identified as 2,4,6-trichloro-3-hydroxy-5-methoxy-toluene (**6**; Monde et al., 1998), *α*-acetylorcinol (**7**; Leyte-Lugo et al., 2020), *O*-methylcurvulinic acid (**8**; Ying et al., 2014), (*S*)-5,7-dichloro-6-methoxy-2-methyl-2,3-dihydrobenzofuran-4-carboxylic acid (**9**; Richardson et al., 2015), pericochlorosin A (**10**; Liu et al., 2020), palmaerones F and G (**11** and **12**; Zhao et al., 2018), 5-chloro-6-hydroxymellein (**13**; Krohn et al., 1997), (*R*)-6-hydroxymellein (**14**; Quang et al., 2013), 3-methyl-6-hydroxy-8-methoxy-3,4-dihydroisocoumarin (**15**; Dethoup et al., 2007), (22*E*, 24*R*)-ergosta-5,7,22-trien-3*β*-ol (**16**; Liu et al., 2012), uracil (**17**; Liu et al., 2009), cyclohelminthol I (**18**; Honmura et al., 2016), and kojic acid (**19**; Li et al., 2003), respectively, by comparison of their NMR data with those reported ones. Besides, the X-ray crystal structures of **4** (CDCC 2133225, Figure 3) and **12** (CDCC 2133224, Figure 3) were reported herein for the first time with flack parameters of 0.005 (5) and 0.000 (16), respectively, which further unambiguously confirmed their absolute configurations.

### Compounds **1**, **4**, **7**, **9**, **13**, **15**, **16**, and **19** inhibited LPS-induced inflammation in RAW264.7 macrophages *via* promoting M2-type macrophage polarization

**Figure 4:**
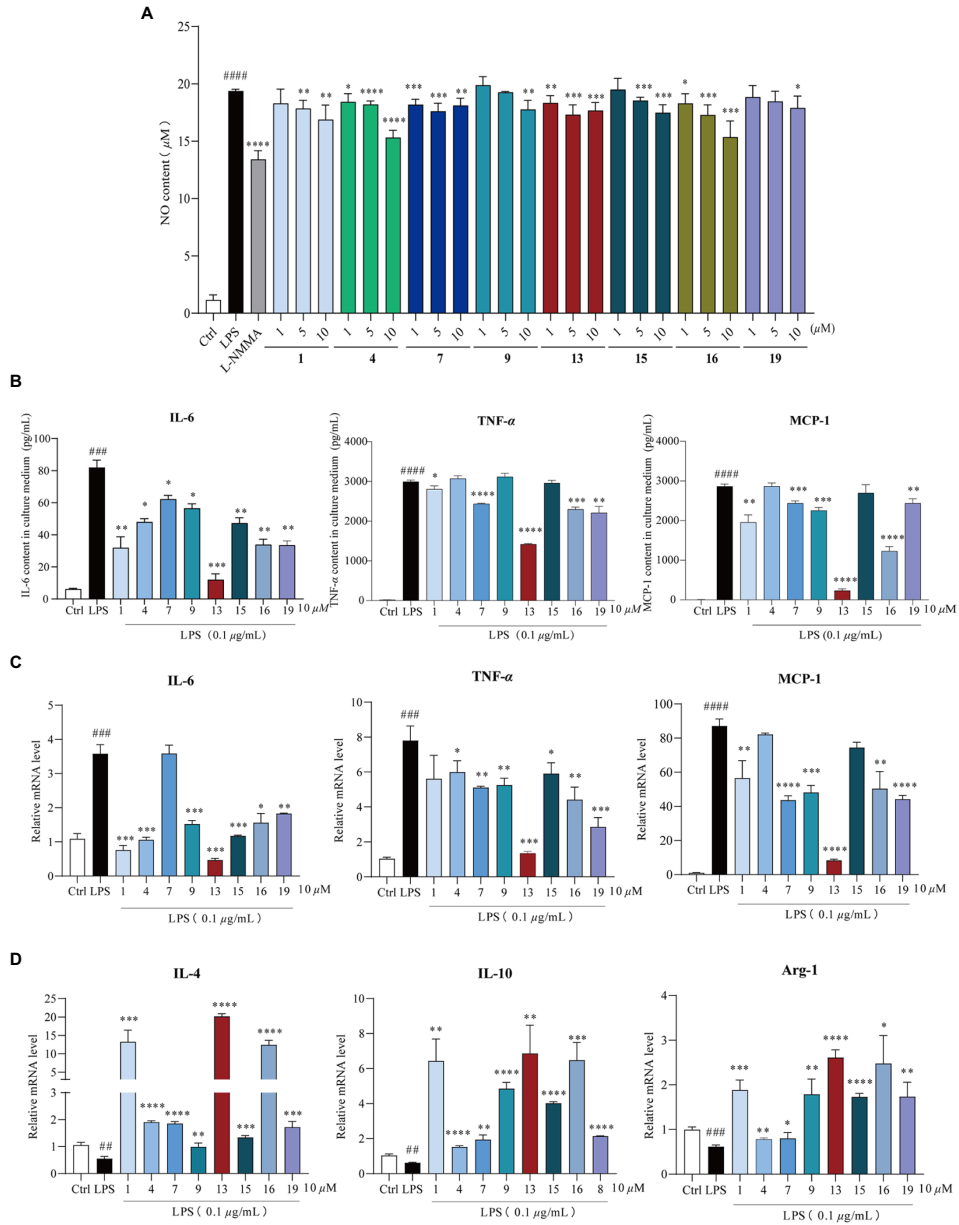
Anti-inflammation activity of compounds **1**, **4**, **7**, **9**, **13**, **15**, **16**, and **19**. Cells were exposed to 0.1 μg/ml lipopolysaccharide (LPS) and co-treated with **1**, **4**, **7**, **9**, **13**, **15**, **16**, and **19** (1, 5, and 10 μM) for 24 h, respectively. **(A)** Nitric oxide (NO) production in the supernatant after 24 h of LPS induction, *n* = 5. L-NMMA was used as the positive control; **(B)** compounds **1**, **4**, **7**, **9**, **13**, **15**, **16**, and **19** reduced LPS-induced IL-6, TNF-*α*, and MCP-1 release, *n* = 3; **(C)** qPCR analysis of M1-type macrophage genes (IL-6, TNF-*α*, and MCP-1) normalized by *β*-actin, *n* = 3; **(D)** qPCR analysis of M2-type macrophage genes (IL-4, IL-10, and Arg-1) normalized by *β*-actin, *n* = 3. All data were presented as the mean ± SD of three independent experiments. ns, *p* > 0.05, ^*^p < 0.05, ^**^p < 0.01, ^***^p < 0.005, ^****^p < 0.001 vs. LPS group; ^##^*p* < 0.01, ^###^*p* < 0.005, ^####^*p* < 0.001 vs. control group, *n* = 3. *p* value was assessed by two-tailed Student’s *t*-test.

These compounds were primarily evaluated with no cytotoxicity against RAW264.7 cells (Supplementary Figure 25). Next, RAW264.7 cells were exposed to 0.1 μg/ml LPS and were co-treated with the same dose of the compounds (10 μM) for 24 h to evaluate their anti-inflammation activities. Compounds **1**, **4**, **7**, **9**, **13**, **15**, **16**, and **19** suppressed LPS-induced NO accumulation in a dose-dependent manner (Figure 4A), while others showed no reduction (Supplementary Figure 26). By comparison with the structural characteristics among them, a preliminary structure–activity relationship is discussed. The chlorine atom at C-5 and hydrogen atom at C-7, together with hydroxy group at C-8 in **13**, would probably increase the inflammatory activity, and the chlorine atom at C-7 may decrease the activity. Moreover, compounds **1**, **4**, **7**, **9**, **13**, **15**, **16**, and **19** suppressed both the mRNA expression and release of pro-inflammatory factors, including IL-6, TNF-*α* (Li et al., 2021), and MCP-1 (Matsuzawa-Ishimoto et al., 2018; Figures 4B,C). In addition, anti-inflammatory genes (IL-4, IL-10, and Arg-1; Georgakis et al., 2019) were also significantly upregulated in response to compound treatment (Figure 4D). These investigations indicate that these eight bioactive compounds could block the expression of pro-inflammatory factors *via* promoting the M2-type macrophage polarization to fight against LPS-induced inflammation.

### Compound 13 alleviated the pathological lung injury of LPS-administrated mice

**Figure 5:**
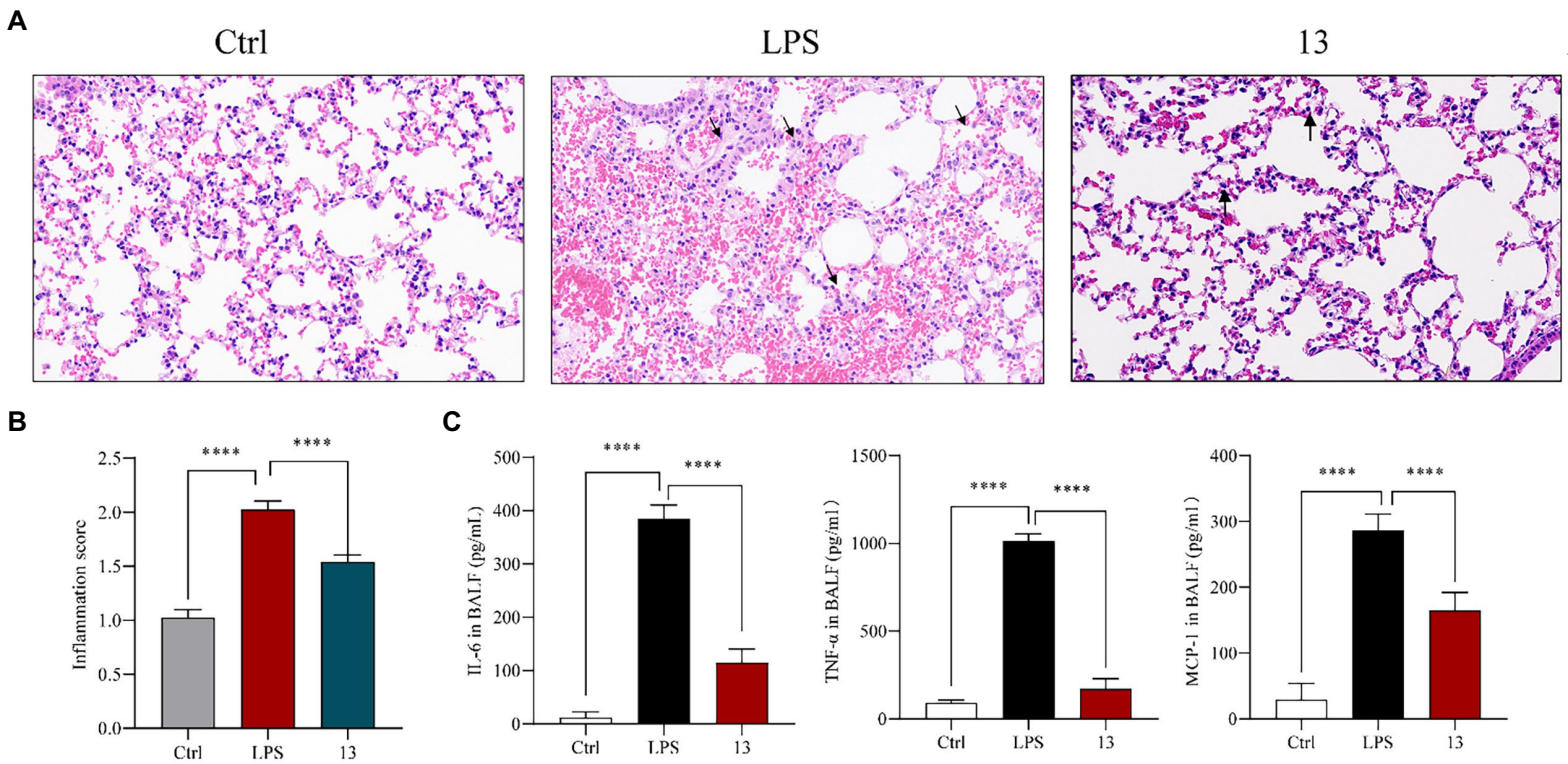
Compound **13** weakens the pathological lung injury of LPS-challenged mice. BALB/c mice were intragastric injected with compound **13** for 24 h before the LPS administration. Mice were killed after 6 h of LPS injection (5 mg/kg), and lung histopathology was performed with *H&E* staining **(A)**. Inflammation score was measured independently by three pathologists blinded to the experiment (**B**, *n* = 6). The levels of anti-inflammation cytokines in BALF were assayed **(C)**. ^****^p < 0.001 vs. control group, *n* = 6. *p* value was assessed by two-tailed Student’s *t*-test.

**Figure 6:**
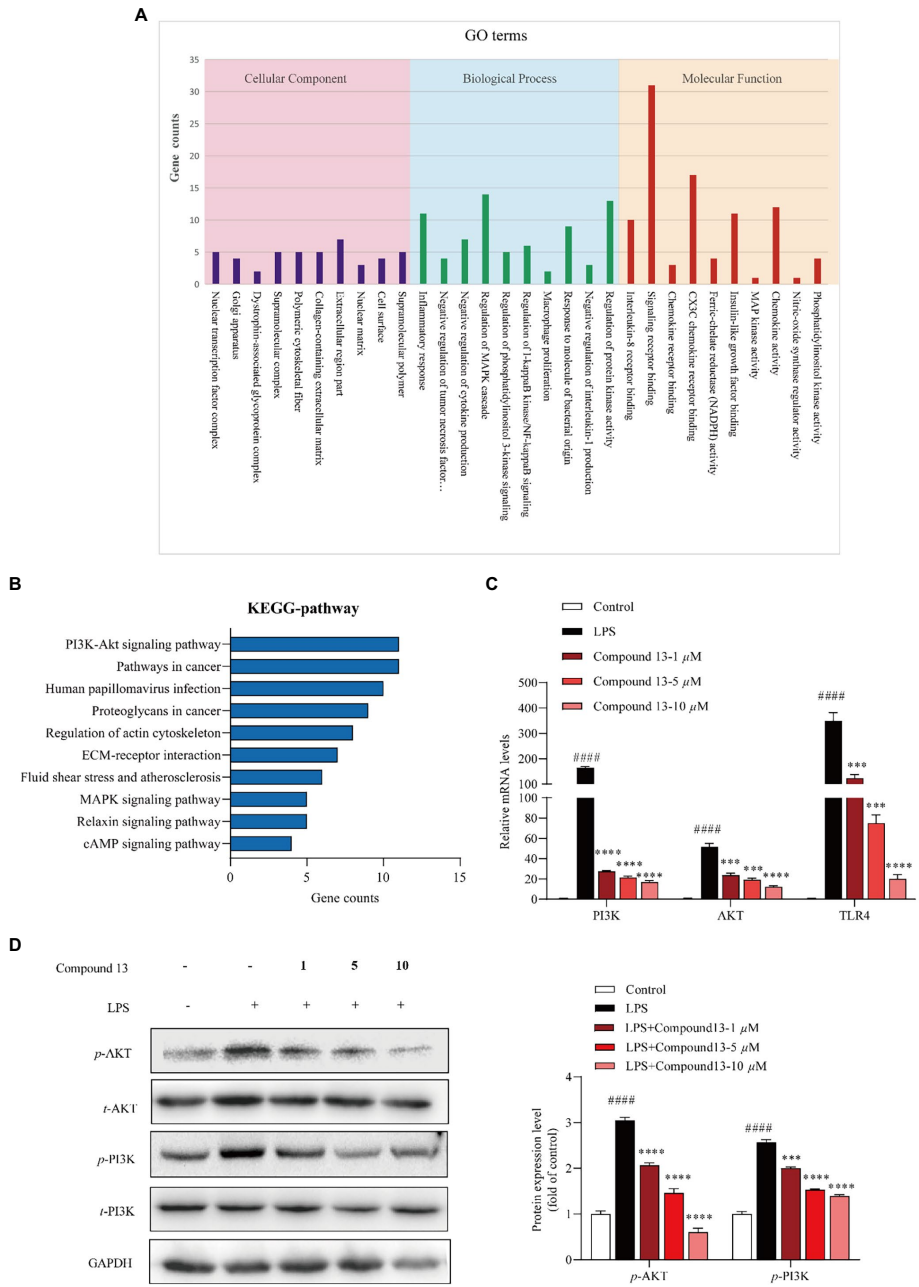
PI3K/AKT signaling pathway was involved in the regulation of LPS-induced inflammation by compound **13** in RAW264.7 macrophages. **(A)** Inflammation-related GO terms enriched from differentially expressed genes (DEGs) between compound **13**-treated and model RAW264.7 macrophages. **(B)** Inflammation-related KEGG pathways enriched from DEGs. **(C)** qPCR analysis of PI3K, AKT, and TLR4 genes normalized by *β*-actin. All data were presented as the mean ± SD of three independent experiments. *p* > 0.05, ns; ^***^p < 0.005, ^****^p < 0.001 vs. LPS group; ^####^*p* < 0.001 vs. control group, *n* = 3. *p* value was assessed by two-tailed Student’s *t*-test. **(D)** Immunoblotting against the PI3K, AKT proteins, *n* = 3. All data were presented as the mean ± SD of three independent experiments. ^***^p < 0.005, ^****^p < 0.001 vs. LPS group; ^####^*p* < 0.001 vs. control group, *n* = 3. *p* value was assessed by two-tailed Student’s *t*-test.

These anti-inflammatory activity results suggested that these marine natural products possessed excellent suppression on nitric oxide and pro-inflammatory cytokines. In addition, compound **13** showed the greatest anti-inflammatory potential among these chlorinated compounds; thus, its inhibitory effect was subsequently inspected *in vivo* (Yang et al., 2020). In brief, BALB/c mice were orally administrated with compound **13** at a dose of 20 mg/kg for 24 h before the LPS treatment, and mice were killed 6 h after intratracheal injection of LPS (5 mg/kg). Lung histopathology was performed with *H&E* staining, which demonstrated that the physiological lung tissue of mice treated with compound **13** was relatively intact with less inflammatory cell infiltration compared with the model group. Besides, the alveoli were clearly delineated and demarcated, and no proliferation of alveolar epithelial cells was observed (Figures 5A,B, as shown by the arrow). These results indicated that compound **13** attenuated the acute lung injury (ALI) caused by LPS-induced inflammation. Furthermore, “cytokine storm” is well characterized in ALI (Chen et al., 2021c). The cytokine detection in the bronchoalveolar lavage fluid (BALF) revealed that the levels of pro-inflammatory factors, such as IL-6, TNF-*α*, and MCP-1, in compound **13** administrated group were generally lower (Figure 6C) than those of the model group. Taken together, our results prompted that compound **13** effectively alleviated inflammation levels in mice with acute lung injury pneumonia (Figure 5C).

### Compound 13 protected RAW264.7 macrophages against LPS-induced inflammation through PI3K/AKT pathway

To further explore the mechanism of compound **13** in ameliorating LPS-induced inflammation, high-throughput sequencing of RNAs from RAW264.7 macrophages was conducted. The results of the GO enrichment analysis indicated that chemokine activity and nitric oxide synthase regulatory activity may play key roles in inflammation induced by LPS (Figure 6A and Supplementary Figure 27). Furthermore, combining the results of the KEGG pathway analysis uncovered that the PI3K/AKT signaling pathway was significantly activated, indicating that it might be a potential key pathway in mediating inflammatory responses in the current cell model (Figure 6B). Consistent with this hypothesis, our subsequent research displayed that compound **13** could effectively restrain the expression levels of PI3K and AKT upregulated by LPS (Figures 6C,D), proving that the PI3K/AKT signaling pathway was involved in this procedure regulated by compound **13**, which is in accordance with another research (Zhong et al., 2016). In this study, we found that compounds **4**, **9**, **13**, and **15** could effectively inhibit the synthesis of NO and overproduction of pro-inflammatory cytokines induced by LPS. Especially, compound **13** also exhibited an outstanding anti-inflammatory activity *in vivo*, suggesting the most active compound.

Mitochondria are key organelles involved in metabolic regulation, and their dysfunction is closely related to metabolic inflammatory diseases. Although the gene expression heat map showed that genes related to mitochondrial function and transcription factors were largely enriched (Supplementary Figure 27), whether they participated in LPS-induced inflammatory response of compound **13** still needs further investigation.

## Conclusion

In summary, the chemical investigation of the mangrove endophytic fungus *Amorosia* sp. SCSIO 41026 resulted in the isolation and identification of 19 secondary metabolites, including three new chlorine-containing fungal metabolites and two new natural products. Our results demonstrated that compounds **1**, **4**, **7**, **9**, **13**, **15**, **16**, and **19** inhibited LPS-induced overproductions of NO and pro-inflammatory cytokines including IL-6, TNF-*α*, and MCP-1 both in mRNA and protein levels with a premise that none of these compounds showed significant cytotoxicity. Among these metabolites, compound **13** was identified as the most active compound with an outstanding ability of anti-LPS-induced inflammation in RAW264.7 macrophages and in ALI mice, probably by inhibiting the PI3K/AKT signaling pathway. Thus, this study demonstrates the potential value of mangrove endophytic fungus as a promising source of bioactive compounds.

## Data availability statement

The data presented in the study are deposited in the NCBI repository, accession number GSE207442 (https://www.ncbi.nlm.nih.gov/sra/PRJNA855018), CDCC Nos: 2133225 and 2133224.

## Ethics statement

The animal study was reviewed and approved by the Medical Ethics Committee of Peking Union Medical College. Written informed consent was obtained from the owners for the participation of their animals in this study.

## Author contributions

XR and CC performed the experiments and wrote the paper. YY, ZX, and XL helped with the analysis of the data. PG, XL, and YL designed and supervised the experiments. All authors contributed to the article and approved the submitted version.

## Funding

This research was funded by CXYJ-2022-01 from the Research Foundation of Capital Institute of Pediatrics (China), Guangdong-Joint Foundation of Shenzhen (2021B1515120046), and the Special Fund for Bagui Scholars of Guangxi (YL).

## Conflict of interest

The authors declare that the research was conducted in the absence of any commercial or financial relationships that could be construed as a potential conflict of interest.

## Publisher’s note

All claims expressed in this article are solely those of the authors and do not necessarily represent those of their affiliated organizations, or those of the publisher, the editors and the reviewers. Any product that may be evaluated in this article, or claim that may be made by its manufacturer, is not guaranteed or endorsed by the publisher.
