# Supplementary material for: Anti-inflammatory compounds from the mangrove endophytic fungus Amorosia sp. SCSIO 41026
Source: Front Microbiol. 2022 Aug 29;13:976399. doi: 10.3389/fmicb.2022.976399 (PMC9533711; doi:10.3389/fmicb.2022.976399)
Supplement: Supplementary file 1 [file Data_Sheet_1.docx]

Supplementary Material
		1       Supplementary Data
The strain’s (Amorosia sp. SCSIO 41026) ITS sequence of the rDNA
TGCTTCCGTAGGGGAACCTGCGGAAGGATCATTACTGCGTGAGGTGGCCCGTGGCCGCCCTCGTCCTTGCCATATTTTTTGGGTACCTTTTGTTTCCTTAGCAGGTCCGCCTGCTCTGGTGCGGGTGCGGCCCCTTCACAGGGCGTGCGGCCCGCGCCGGAAGCGGCGGCCTCTGAGGACCCCTAAACCTTTTGTAAACGCTGTAGTCGTCTGAGAAAACAAAAACGAATTAAAACTTTCAACAATGGATCTCTTGGTTCTGGCATCGATGAAGAACGCAGCAAAATGCGATAAGTAGTGTGAATTGCAGAATTCCGTGAATCATCGAATCTTTGAACGCACATTGCGCCCTTTGGTATTCCTTAGGGCATGCCTGTTCGAGCGTCATTTCAACCCTCAAGCTCCGCTTGGTGTTGGGCGTCTGTCCCGCCGTCACGGCGAGGACTCGCCTCAAATCGATTGGCGGCCTCCCGGCAGCTCAACGCGCAGCAGACTTGCGCCGCGTGCAGCCAGCGGGGGGTTCAACCATCAAGACTTCTCCAGTTTGACCTCGGATCAGGTAGGGATACCCGCTGAACTTAAGCATATCATAGGCCGGAGGAA
The physicochemical data of the known compounds 4–19
7-Chloro-3,4-dihydro-6,8-dihydroxy-3-methylisocoumarine (4) (Henderson et al., 1982): white, amorphous solid; [α]25 D −37 (c 0.01, CH3OH); 1H NMR (700 MHz, DMSO-d6) δ 11.74 (1H, s, 8-OH), 11.48 (1H, s, 6-OH), 6.45 (1H, s, H-4), 4.73 (1H, dqd, J = 12.6, 6.3, 3.5 Hz, H-3), 2.95 (1H, dd, J = 16.1, 3.5 Hz, H-4a), 2.83 (1H, ddd, J = 16.1, 11.2, 1.4 Hz, H-4b), 1.39 (3H, dd, J = 6.3, 2.1 Hz, H3-8); 13C NMR (175 MHz, DMSO-d6) δ 169.5 (C-1), 160.0 (C-8), 158.8 (C-6), 139.6 (C-4a), 106.5 (C-5), 105.1 (C-7), 100.8 (C-8a), 75.8 (C-3), 33.3 (C-4), 20.2 (C-9).
2,4-Dichloro-3-hydroxy-5-methoxy-toluene(5) (Calam et al., 1939): clear colorless needle crystal; 1H NMR (500 MHz, DMSO-d6) δ 9.86 (1H, s, 1-OH), 6.69 (1H, s, H-6), 3.80 (3H, s, H3-7), 2.29 (3H, s, H3-8); 13C NMR (125 MHz, DMSO-d6) δ 153.6 (C-3), 149.6 (C-5), 135.0 (C-1), 114.6 (C-2), 107.7 (C-4), 105.5 (C-6), 56.2 (C-7), 20.3 (C-8).
2,4,6-Trichloro-3-hydroxy-5-methoxytoluene (6) (Monde et al., 1998): colorless oil; 1H NMR (500 MHz, DMSO-d6) δ 3.78 (3H, s, H3-7), 2.40 (3H, d, J = 1.0 Hz, H3-8); 13C NMR (125 MHz, DMSO-d6) δ 151.0 (C-3), 149.1 (C-5), 133.4 (C-1), 119.2 (C-2), 118.8 (C-6), 115.2 (C-4), 60.3 (C-7), 18.0 (C-8).
α-Acetylorcinol (7) (Leyte-Lugo et al., 2020): amber oil; 1H NMR (500 MHz, DMSO-d6) δ 9.18 (2H, s, 3, 5-OH), 6.08 (3H, t, J = 2.0 Hz, H-4), 6.03 (2H, d, J = 2.0 Hz, H-2, 6), 3.49 (2H, s, H2-7), 2.06 (3H, s, H3-9); 13C NMR (125 MHz, DMSO-d6) δ 206.1 (C-8), 158.4 (C-3, 5), 136.5 (C-1), 107.5 (C-2, 6), 100.9 (C-4), 50.1 (C-7), 29.1 (C-9).
O-Methylcurvulinic acid (8) (Ying et al., 2014): amber oil; 1H NMR (500 MHz, DMSO-d6) δ 12.12 (1H, s, 8-COOH), 10.08 (1H, s, 3-OH), 9.78 (1H, s, 5-OH), 6.27 (1H, d, J = 2.0 Hz, H-4), 6.12 (1H, d, J = 2.0 Hz, H-6), 3.49 (2H, s, H-7), 2.38 (3H, s, H3-10); 13C NMR (125 MHz, DMSO-d6) δ 202.5 (C-9), 172.4 (C-8), 159.5 (C-5), 158.1 (C-3), 136.1 (C-1), 119.9 (C-2), 110.4 (C-6), 101.4 (C-4), 39.5 (C-7), 32.0 (C-10).
Kojic acid (8) (Li et al., 2003): colorless needles; 1H NMR (500 MHz, DMSO-d6) δ 8.10 (1H, s, H-6), 6.42 (1H, s, H-3), 4.37 (2H, s, H2-7); 13C NMR (125 MHz, DMSO-d6) δ 173.9 (C-4), 168.4 (C-2), 145.9 (C-5), 139.5 (C-6), 110.0 (C-3), 59.7 (C-7).
(R)-5,7-Dichloro-6-methoxy-2-methyl-2,3-dihydrobenzofuran-4-carboxylic acid (9) (Richardson et al., 2015): white, amorphous solid; [α]25 D −41 (c 0.01, CH3OH); 1H NMR (700 MHz, DMSO-d6) δ 4.56 (1H, dqd, J = 12.5, 6.3, 3.5 Hz, H-3), 3.82 (3H, s, H3-10), 3.14 (1H, m, H-4a), 2.76 (1H, dd, J = 16.8, 11.2 Hz, H-4b), 1.39 (3H, d, J = 6.3 Hz, H3-9); 13C NMR (175 MHz, DMSO-d6) δ 160.5 (C-1), 157.4 (C-8), 154.5 (C-6), 138.7 (C-4a), 116.1 (C-8a), 115.3 (C-5), 111.4 (C-7), 72.9 (C-3), 61.5 (C-10), 32.9 (C-4), 20.2 (C-9).
Pericochlorosin A (10) (Liu et al., 2020): white, amorphous solid; [α]25 D −33 (c 0.01, CH3OH);1H NMR (500 MHz, DMSO-d6) δ 11.87 (1H, s, 8-OH), 4.76 (1H, dqd, J = 12.5, 6.0, 3.0 Hz, H-3), 3.18 (1H, m, H-4a), 2.82 (1H, dd, J = 17.0, 11.5 Hz, H-4b), 1.44 (3H, d, J = 6.3 Hz, H3-9), 1.23 (1H, s); 13C NMR (125 MHz, DMSO-d6) δ 169.1 (C-1), 157.4 (C-8), 156.1 (C-6), 136.8 (C-4a), 111.1 (C-5), 107.3 (C-7), 101.4 (C-8a), 75.1 (C-3), 31.5 (C-4), 20.2 (C-9).
Palmaerone F (11) (Zhao et al., 2018): white, amorphous solid; [α]25 D −40 (c 0.01, CH3OH); 1H NMR (500 MHz, DMSO-d6) δ 6.51 (1H, d, J = 3.0 Hz, H-7), 4.41 (1H, dqd, J = 12.5, 6.5, 2.5 Hz, H-3), 3.70 (3H, s, H3-10), 3.08 (1H, dd, J = 16.5, 3.0 Hz, H-4a), 2.62 (1H, dd, J = 16.5, 11.3 Hz, H-4b), 1.35 (3H, d, J = 6.5 Hz, H3-9); 13C NMR (125 MHz, DMSO-d6) δ 155.7 (C-1), 153.7 (C-8), 151.2 (C-6), 133.2 (C-4a), 102.3 (C-5), 97.4 (C-8a), 90.5 (C-7), 65.4 (C-3), 47.0 (C-10), 24.9 (C-4), 11.3 (C-9).
Palmaerone G (12) (Zhao et al., 2018): colorless needle crystal; [α]25 D −48 (c 0.01, CH3OH); 1H NMR (500 MHz, DMSO-d6) δ 11.34 (1H, s, 6-OH), 6.70 (1H, s, H-5), 4.53 (1H, dqd, J = 12.0, 6.0, 2.5 Hz, H-3), 3.81 (3H, s, H3-10), 2.91 (1H, dd, J = 16.5, 3.0 Hz, H-4a), 2.78 (1H, dd, J = 16.5, 11.5 Hz, H-4b), 1.36 (3H, d, J = 6.5 Hz, H3-9); 13C NMR (125 MHz, DMSO-d6) δ 161.0 (C-1), 159.0 (C-8), 158.4(C-6), 141.2 (C-4a), 114.2 (C-7), 110.3 (C-5), 110.3 (C-8a), 73.5 (C-3), 61.1 (C-10), 34.8 (C-4), 20.2 (C-9).
5-Chloro-6-hydroxymellein (13) (Krohn et al., 1997): white, amorphous solid; [α]25 D −38 (c 0.01, CH3OH); 1H NMR (700 MHz, DMSO-d6) δ 11.57 (1H, s, 8-OH), 11.22 (1H, s, 6-OH), 6.45 (1H, d, J = 0.7 Hz, H-7), 4.72 (1H, dqd, J = 12.6, 6.3, 3.5 Hz, H-3), 3.17 (1H, dd, J = 16.8, 3.5 Hz, H-4b), 2.78 (1H, ddd, J = 16.8, 11.2, 0.7 Hz, H-4a), 1.43 (3H, d, J = 6.3 Hz, H3-9); 13C NMR (175 MHz, DMSO-d6) δ 169.1 (C-1), 161.6 (C-8), 160.1 (C-6), 139.1 (C-4a), 109.9 (C-5), 101.7 (C-7), 101.0 (C-8a), 74.8 (C-3), 31.7 (C-4), 20.3 (C-9).
(R)-6-Hydroxymellein (14) (Quang et al., 2013): white, amorphous solid; [α]25 D −35 (c 0.01, CH3OH); 1H NMR (700 MHz, DMSO-d6) δ 11.12 (1H, s, 8-OH), 6.23 (1H, d, J = 2.1 Hz, H-7), 6.18 (1H, d, J = 2.1 Hz, H-5),4.68 (1H, dqd, J = 12.6, 6.3, 3.5 Hz, H-3), 2.84 (2H, m, H-4), 1.38 (3H, d, J = 6.3 Hz, H3-9); 13C NMR (175 MHz, DMSO-d6) δ 169.5 (C-1), 164.5 (C-8), 163.4 (C-6), 142.3 (C-4a), 106.8 (C-5), 100.9 (C-8a), 100.1(C-7), 75.6 (C-3), 33.8 (C-4), 20.3 (C-9).
3-Methyl-6-hydroxy-8-methoxy-3,4-dihydroisocoumarin (15) (Dethoup et al., 2007): white, amorphous solid; [α]25 D −51 (c 0.01, CH3OH); 1H NMR (500 MHz, DMSO-d6) δ 6.37 (1H, d, J = 2.1 Hz, H-7), 6.26 (1H, d, J = 2.1 Hz, H-5), 4.41 (1H, ddp, J = 12.5, 6.5, 3.0 Hz, H-3), 3.74 (3H, s, H-10), 2.81 (1H, dd, J = 16.0, 3.0 Hz, H-4a), 2.72 (1H, d, J = 11.0 Hz, H-4b), 1.31 (3H, d, J = 6.5 Hz, H-9); 13C NMR (125 MHz, DMSO-d6) δ 162.9 (C-8), 162.8 (C-1), 161.2 (C-6), 143.9 (C-4a), 106.2 (C-5), 104.5 (C-8a), 98.4 (C-7), 72.9(C-3), 55.5 (C-10), 35.5 (C-4), 20.3 (C-9).
(22E,24R)-ergosta-5,7,22-trien-3β-ol (16) (Liu et al., 2012): colorless needle crystal; 1H NMR (500 MHz, Chloroform-d) δ5.56 (1H, dd, J = 5.5, 2.5 Hz, H-6), 5.38 (1H, m, H-7), 5.19 (2H, dd, J = 12.5, 7.5 Hz, H-22, 23), 3.64 (1H, m, H-3), 2.46 (1H, ddd, J = 14.5, 5.0, 2.5 Hz, H-4α), 2.28 (1H, m, H-4β), 1.03 (3H, d, J = 6.5 Hz, H3-21), 0.94 (3H, s, H3-19), 0.91 (3H, d, J = 7.0 Hz, H3-28), 0.82 (6H, m, H3-26, 27), 0.62 (3H, s, H3-18); 13C NMR (125 MHz, Chloroform-d) δ 141.5 (C-8), 139.9 (C-5), 135.7 (C-22), 132.1 (C-23), 119.7 (C-6), 116.4 (C-7), 70.6 (C-3), 55.8 (C-17), 54.7 (C-14), 46.4 (C-9), 43.0 (C-24), 40.9 (C-13), 39.2 (C-12), 38.5 (C-1), 37.2 (C-10), 33.2 (C-25), 32.1 (C-2), 28.4 (C-16), 23.1 (C-15), 21.2 (C-11, 21), 20.1 (C-27), 19.8 (C-26), 17.8 (C-28), 16.4 (C-19), 12.2 (C-18).
Uracil (17) (Liu et al., 2009): white powder; 1H NMR (500 MHz, DMSO-d6) δ 11.02 (1H, s, NH-3), 10.83 (1H, s, NH-1), 7.39 (1H, d, J = 7.5 Hz, H-6), 5.44 (1H, dd, J = 7.5, 2.0 Hz, H-5); 13C NMR (125 MHz, DMSO-d6) δ 164.4 (C-4), 151.6 (C-2), 142.2 (C-6), 100.3 (C-5).
Cyclohelminthol I (18) (Honmura et al., 2016): white powder; [α]25 D −18 (c 0.01, CH3OH); 1H NMR (500 MHz, DMSO-d6) δ 6.72 – 6.59 (1H, m, H-7), 6.42 (1H, dd, J = 16.0, 2.0 Hz, H-6), 5.97 (1H, s, H-2), 4.91 (1H, d, J = 6.0 Hz, H-4), 2.70 (1H, d, J = 6.5 Hz, H-5a), 2.66 (1H, d, J = 6.5 Hz, 1-OH), 2.11 (1H, dd, J = 18.2, 1.8 Hz, H-5b), 1.88 (3H, dd, J = 7.0, 1.5 Hz, H-8); 13C NMR (125 MHz, DMSO-d6) δ 205.5 (C-1), 172.6 (C-3), 138.5 (C-7), 127.5 (C-2), 125.7 (C-6), 68.3 (C-4), 45.4 (C-5), 19.0 (C-8).
Kojic acid (19) (Li et al., 2003): colorless needles; 1H NMR (500 MHz, DMSO-d6) δ 8.10 (1H, s, H-6), 6.42 (1H, s, H-3), 4.37 (2H, s, H2-7); 13C NMR (125 MHz, DMSO-d6) δ 173.9 (C-4), 168.4 (C-2), 145.9 (C-5), 139.5 (C-6), 110.0 (C-3), 59.7 (C-7).
Calam, C. T.; Oxford, A. E. (1939). Halogen derivatives of the methyl ethers of orcinol, p-orsellinic acid and phloroglucinolcarboxylic acid. J. Chem. Soc, 280–284. doi:10.1039/jr9390000280
Dethoup, T.; Manoch, L.; Kijjoa, A.; Pinto, M.; Gales, L.; Damas, A. M.; Silva, A. M. S.; Eaton, G.; Herz, W., (2007). Merodrimanes and other constituents from Talaromyces thailandiasis. J. Nat. Prod, 70, 1200–1202. doi:10.1021/np0680578 
Henderson, G. B.; Hill, R. A. (1982). Synthesis of chlorinated isocoumarin derivatives. J. Chem. Soc. Perkin Trans, 1, 1111–1115. doi:10.1039/P19820001111
Honmura, Y.; Uesugi, S.; Maeda, H.; Tanaka, K.; Nehira, T.; Kimura, K. I.; Okazaki, M.; Hashimoto, M. (2016). Isolation, absolute structures, and biological properties of cyclohelminthols I–IV from Helminthosporium velutinum yone96. Tetrahedron, 72, 1400–1405. doi: 10.1002/chin.201625219
Krohn, K.; Bahramsari, R.; Flsrke, U.; Ludewig, K.; Kliche-Spory, C.; Michel, A.; Aust, H. J.; Draeger, S.; Schulz, B.; Antus, S. (1997). Dihydroisocoumarins from fungi: isolation, structure elucidation, circular dichroism and biological activity. Phytochemistry, 45, 313–320. doi:10.1016/s0031-9422(96)00854-0 
Leyte-Lugo, M.; Richomme, P.; Poupard, P.; Pena-Rodriguez, L. M. (2020). Identification and quantification of a phytotoxic metabolite from Alternaria dauci. Molecules, 25, 2108–2112. doi:10.3390/molecules25174003 
Li, X.; Jeong, J. H.; Lee, K. T.; Rho, J. R.; Son, B. W. (2003). γ-Pyrone derivatives, kojic acid methyl ethers from a marine-derived Fungusaltenaria sp. Arch. Pharm. Res, 26, 532–534. doi:10.1007/BF02976876 
Liu J.; Chen, M.; Chen, R.; Xie, K.; Chen, D.; Si, S.; Dai, J. (2020). Three new compounds from endophytic fungus Periconia sp. F-31. J. Pharm, 29, 244–251. doi:org/10.5246/jcps.2020.04.023 
Liu, R. H.; Luo, H.; Li, Y. L.; Ming, Y.; Xu, X. K.; Li, H. L. (2009). N-containing compounds from the traditional Chinese medicine ChanSu. Chem. Nat. Compd, 45, 599–600. doi: 10.1007/s10600-009-9380-4
Liu, T.; Zhang, L.; Li, Z.; Wang, Y.; Tian, L.; Pei, Y.; Hua, H. (2012). A new sulfo-xanthone from the marine-derived fungus penicillium sacculum. Chem. Nat. Compd, 48, 771–773. doi: 10.1080/14786419.2015.1101693 
Monde, K.; Satoh, H.; Nakamura, M.; Tamura, M.; Takasugi, M. (1998). Organochlorine compounds from a terrestrial higher plant: Structures and origin of chlorinated orcinol derivatives from diseased bulbs of Lilium maximowiczii. J. Nat. Prod, 61, 913–921. doi:10.1021/np980062r
Quang, D. N.; Lam, D. M.; Hanh, N. T. H.; Que, D. D. (2013). Cytotoxic constituents from the fungus Daldinia concentrica (Xylariaceae). Nat. Prod. Res, 27, 486–490. doi:10.1080/14786419.2012.698411 
Richardson, S. N.; Nsiama, T. K.; Walker, A. K.; McMullin, D. R.; Miller, J. D. (2015). Antimicrobial dihydrobenzofurans and xanthenes from a foliar endophyte of Pinus strobus. Phytochemistry, 117, 436–443. doi:10.1016/j.phytochem.2015.07.009
Ying, Y. M.; Zhang, L. W.; Shan, W. G.; Zhan, Z. J. (2014). Secondary metabolites of Peyronellaea sp. XW-12, an endophytic fungus of Huperzia serrata. Chem. Nat. Compd, 50, 723–725. doi:10.1007/s10600-014-1063-0
Zhao, M.; Yuan, L. Y.; Guo, D. L.; Ye, Y.; Da-Wa, Z. M.; Wang, X. L.; Ma, F. W.; Chen, L.; Gu, Y. C.; Ding, L. S.; Zhou, Y. (2018). Bioactive halogenated dihydroisocoumarins produced by the endophytic fungus Lachnum palmae isolated from Przewalskia tangutica. Phytochemistry, 148, 97–103. doi:10.1016/j.phytochem.2018.01.018
		Supplementary Figures and Tables
		2.1   Supplementary Figures

Supplementary Figure 1. 1H NMR spectrum of chlomophenol A (1) in DMSO-d6.

Supplementary Figure 2. 13C NMR spectrum of chlomophenol A (1) in DMSO-d6.

Supplementary Figure 3. HSQC spectrum of chlomophenol A (1) in DMSO-d6.

Supplementary Figure 4. HMBC spectrum of chlomophenol A (1) in DMSO-d6.

Supplementary Figure 5. 1H–1H COSY spectrum of chlomophenol A (1) in DMSO-d6.

Supplementary Figure 6. HRESIMS spectrum of chlomophenol A (1).

Supplementary Figure 7. IR spectrum of chlomophenol A (1).

Supplementary Figure 8. UV spectrum of chlomophenol A (1) in MeOH.

Supplementary Figure 9. 1H NMR spectrum of chlomophenol B (2) in DMSO-d6.


Supplementary Figure 10. 13C NMR spectrum of chlomophenol B (2) in DMSO-d6.

Supplementary Figure 11. HSQC spectrum of chlomophenol B (2) in DMSO-d6.

Supplementary Figure 12. HMBC spectrum of chlomophenol B (2) in DMSO-d6.

Supplementary Figure 13. 1H–1H COSY spectrum of chlomophenol B (2) in DMSO-d6.

Supplementary Figure 14. HRESIMS spectrum of chlomophenol B (2).

Supplementary Figure 15. IR spectrum of chlomophenol B (2).

Supplementary Figure 16.  UV spectrum of chlomophenol A (1) in MeOH.

Supplementary Figure 17. 1H NMR spectrum of chlomophenol C (3) in DMSO-d6.

Supplementary Figure 18.  13C NMR spectrum of chlomophenol C (3) in DMSO-d6.

Supplementary Figure 19.  HSQC spectrum of chlomophenol C (3) in DMSO-d6.

Supplementary Figure 20. HMBC spectrum of chlomophenol C (3) in DMSO-d6.

Supplementary Figure 21.  HRESIMS spectrum of chlomophenol C (3).

Supplementary Figure 22. IR spectrum of chlomophenol C (3).

Supplementary Figure 23. UV spectrum of chlomophenol C (3) in MeOH.

Supplementary Figure 24. Calibration curves of nitro oxide detection. Standard substance was diluted to 0, 1, 2, 5, 10, 20, 40, 60, 100 μM with full cell culture medium (high-glucose DMEM+10% FBS+1% PS), and the wells with PBS was determined as negative control in the meanwhile. The components were added in sequence according to the instructions and read at 540 nm with a microplate reader. Graphpad Prism software was used to plot the calibration curves for subsequent experiments.


Supplementary Figure 25. Compounds 1−19 showed no cytotoxicity to RAW264.7 cells at the dose of 10 μM. Cells were exposed to compound 1−19 (10 μM) for 24 hours, respectively. All data are presented as the mean ± SD of three independent experiments, n=6.

Supplementary Figure 26. Nitro oxide production of RAW264.7 cells. Cells were exposed to 0.1 μg/mL LPS and co-treated with compound 1−19 (10 μM) for 24 hours (n=5). All data are presented as the mean ± SD of three independent experiments. ns, P>0.05, *P<0.05, **P<0.01, ***P<0.005 vs. model group; ####P<0.001 vs. control group. P value was assessed by two-tailed Student’s t-test.

Supplementary Figure 27. Heatmap of differentially expressed genes (DEGs) between Compound 13-treated and model RAW264.7 macrophages. 

Supplementary Figure 28. Animal program protocol. Mice were randomly divided into 3 groups (n = 6), the control group (Group B), the model group (5 mg/kg LPS) (Group M), and LPS (5 mg/kg) + compound 13 (20 mg/kg) group (Group A). After 6 h of LPS administration, animals were sacrificed for the bronchoalveolar lavage fluid (BALF) and lung tissues collection.

		Supplementary Tables
Table S1. Primers used in qPCR.
Name	Species	Forward (5'-3')	Reverse (5'-3')
IL-6	Mice	CCGGAGAGGAGACTTCACAG	TGGTCTTGGTCCTTAGCCAC
Tnf - ɑ	Mice	GACCCTCACACTCAGATCAT	TTGAAGAGAACCTGGGAGTA
IL-4	Mice	GGTCTCAACCCCCAGCTAGT	GCCGATGATCTCTCTCAAGTGAT
Arg-1	Mice	TGTCCCTAATGACAGCTCCTT	GCATCCACCCAAATGACACAT
IL-10	Mice	CTTACTGACTGGCATGAGGATCA	GCAGCTCTAGGAGCATGTGG
Mcp-1	Mice	TTAAAAACCTGGATCGGAACCAA	GCATTAGCTTCAGATTTACGGGT
Tlr4	Mice	GCCTTTTCTGGACTATCAAG	AATTTGAAAGATTGGATAAG
Pi3k	Mice	CAAGGTCTCACTTCTGGATCAAGTG	CCTATTTGCCTCTGTCACCTTG
Akt	Mice	CACGAAGCAGGCAGGGCCAA	ACCTCCCGCCCCATTTCTCAT
β-actin	Mice	ACACTGTGCCCATCTACGAG	CAGCACTGTGTTGGCATAGAG

Table S2. Cytokines detection by Ella kit in culture supernatant from Protein Simple Company.
Sample	IL-6	TNF-α	MCP-1
Control	6.14 ± 0.51	19.63 ± 0.32	5.33 ± 0.54
Model	81.89 ± 4.69###	2994.75 ± 37.99####	2859.88 ± 59.15####
1	31.93 ± 6.81**	2804.83 ± 82.35*	1954.73 ± 188.6**
4	47.93 ± 2.12*	3070.88 ± 73.78	2863.79 ± 84.98
7	62.18 ± 2.37*	2434.58 ± 17.08****	2433.36 ± 60.32***
9	56.5 ± 2.79**	3115.79 ± 90.34	2251.79 ± 79.09***
13	11.98 ± 3.68***	1417.5625 ± 17.77****	230.30 ± 43.72****
15	47.24 ± 3.4**	2955.88 ± 67.92	2694.73 ± 212.12
16	33.82 ± 3.38**	2297.5 ± 55.15**	1225.52 ± 116.47****
19	33.5 ± 2.73**	2207.56 ± 165.90**	2434.64 ± 116.71**

RAW264.7 cells were exposed to 0.1 μg/mL LPS and co-treated with compound 1, 4, 7, 9, 13, 15, 16 and 19 (10 μM) for 24 hours (n=5). All data are presented as the mean ± SD of three independent experiments. ns, P>0.05, *P<0.05, **P<0.01, ***P<0.005 vs. model group; ####P<0.001 vs. control group. P value was assessed by two-tailed Student’s t-test.
